# Functional localization of visual motion area FST in humans

**DOI:** 10.1162/imag_a_00578

**Published:** 2025-05-16

**Authors:** Puti Wen, Rania Ezzo, Lowell W. Thompson, Ari Rosenberg, Michael S. Landy, Bas Rokers

**Affiliations:** Psychology, New York University Abu Dhabi, Abu Dhabi, United Arab Emirates; Department of Psychology, New York University, New York, NY, United States; Department of Neuroscience, Perelman School of Medicine, University of Pennsylvania, Philadelphia, PA, United States; Department of Neuroscience, School of Medicine and Public Health, University of Wisconsin-Madison, Madison, WI, United States; Center for Neural Science, New York University, New York, NY, United States

**Keywords:** middle temporal area, fundus of the superior temporal sulcus, 3D motion, binocular vision, functional localization, MT complex

## Abstract

The fundus of the superior temporal sulcus (FST) in macaque monkeys is implicated in the processing of complex motion signals, yet a human homolog remains elusive. To better understand the neural mechanisms underlying the analysis of complex motion signals in humans, it is crucial to understand if and where a region homologous to FST exists. Here, we considered potential localizers and evaluated their effectiveness in delineating putative FST (pFST) from two nearby motion-sensitive areas, hMT and MST, in humans. Nine participants underwent fMRI scanning sessions with 2D and 3D motion localizers, as well as population receptive field (pRF) mapping. We observed consistent anterior and inferior activation relative to hMT and MST in response to stimuli that contained coherent 3D, but not 2D, motion. Motion opponency and myelination measures further validated the functional and structural distinction between pFST and hMT/MST. At the same time, standard pRF mapping techniques that reveal the visual field organization of hMT/MST proved suboptimal for delineating pFST. Our findings provide confirmatory evidence for the existence of a functional homolog of macaque area FST in humans, offer a robust framework for localizing pFST, and underscore the area’s distinct role in visual motion processing.

## 1 Introduction

Much of our understanding of the processing of visual motion is based on work with macaque monkeys. In that species, several visual areas have been identified in the dorsal pathway that are motion selective. These areas include the middle temporal area (MT), the medial superior temporal area (MST), and the fundus of the superior temporal sulcus (FST). Much of the neuroimaging work investigating motion processing in humans has aggregated these areas into a single ‘MT complex’, originally coined byDeYoe et al. (1996)due to the challenges associated with the precise delineation of subregions within this motion-selective cluster in the context of fMRI. While prior work has clearly delineated areas MT and MST in humans based on several criteria, including structural landmarks, myelination, retinotopic organization, and functional specialization (Annese et al., 2005;Dumoulin et al., 2000;Huk et al., 2002;Kolster et al., 2010;Tootell et al., 1995), the case for FST has been much less clear. In macaques, FST processes complex motion signals such as structure from motion, transparent motion, and 3D motion toward/away from the observer (Héjja-Brichard et al., 2020;Mysore et al., 2010;Nelissen et al., 2006;Rosenberg et al., 2008;Thompson et al., 2023;Vanduffel et al., 2002). To better understand the neural mechanisms underlying such functions in humans, it is therefore important to understand if and where a region homologous to FST exists.

In humans, the MT complex is located in the ascending limb of the inferior temporal sulcus, and includes homologs of MT and MST. The preservation of FST across a range of non-human primate species including macaques, squirrel monkeys, owl monkeys, marmosets, and galagos (Krubitzer & Kaas, 1990;Ungerleider & Desimone, 1986a,1986b) suggests that a homolog could exist in humans as part of the motion-processing system. There are, however, no established methods to localize or verify the existence of a human homolog of FST. Therefore, the present study evaluates the effectiveness of potential functional localizers for human FST and evaluates the case for homology using independent measures, including motion opponency, population receptive field estimates, and myelination.

Macaque FST receives major direct projections from MT (Boussaoud et al., 1990;Krubitzer & Kaas, 1990;Ungerleider & Desimone, 1986a,1986b), a region with an established role in the analysis of visual motion (Albright, 1984;Dubner & Zeki, 1971;Maunsell & Van Essen, 1983a,1983b). Although both MT and FST process motion, they play distinct roles, and there is growing evidence that a primary distinction could be the role of FST in processing complex motion signals that extrapolate beyond retinal signals (Rosenberg et al., 2023). A monkey neuroimaging study showed that FST responded more strongly to 3D motion compared to MT (Héjja-Brichard et al., 2020). This was supported by a recent electrophysiology study which found that 37% of FST neurons but only 8% of MT neurons are selective for toward/away motion (Thompson et al., 2023). Furthermore, while the responses of MT neurons to opponent motion signals are suppressed in humans and macaques (Heeger et al., 1999;Qian & Andersen, 1994), FST neurons often respond similarly to a single direction of motion and stimuli containing opposite directions (Rosenberg et al., 2008). FST is also strongly activated by structure-from-motion stimuli, in which stimulus elements can move in opposite directions (Mysore et al., 2010;Vanduffel et al., 2002). Finally, the involvement of FST in processing looming objects and its role in predicting their impact (Cléry et al., 2020) as well as its contribution to action-related visual processing (Nelissen et al., 2006) further differentiate it from neighboring areas.

Previous work established human homologs of MT and MST using 2D-motion localizers (Huk et al., 2002;Tootell et al., 1995). Both areas also show adaptation to 3D-motion stimuli (Rokers et al., 2009) and direction of motion can be decoded from them as well as areas more anterior (Wen et al., 2023). However, these studies primarily relied on stimuli that contained both 3D and 2D (retinal) motion signals. A prior result, utilizing a stimulus that specified 3D motion in the absence of coherent 2D (retinal) motion signals, identified a region anterior and ventral to human MT/MST (Likova & Tyler, 2007), echoing the location of FST in monkey studies. We reasoned that, based on results in monkeys, the use of a 2D motion localizer that does not contain 3D motion, and a 3D motion localizer that does not contain 2D motion, may dissociate human FST from neighboring MT and MST.

Recent advances in the understanding of the functional properties of macaque FST present an opportunity to establish whether a homolog exists in humans, and if so, a method to reliably and accurately localize the area. To evaluate the case for homology, we present evidence for a putative FST region in humans using several distinct functional and structural MRI metrics. Areas MT/MST are functionally characterized by significant signal suppression to opponent motion in monkeys (Qian & Andersen, 1994) and humans (Heeger et al., 1999). Additionally, MT/MST are structurally characterized by high myelination (Boussaoud et al., 1990;Clarke & Miklossy, 1990;Desimone & Ungerleider, 1986;Van Essen et al., 1981). Area FST, on the other hand, shows little suppression to opponent motion (Rosenberg et al., 2008) and is less myelinated in monkeys (Kaas & Morel, 1993;Krubitzer & Kaas, 1990;Rosa et al., 1993) and possibly humans (Abdollahi et al., 2014;Glasser et al., 2016). By integrating these anatomical and functional characteristics—such as opponent-motion suppression, population receptive field organization, and myelin patterns—we aim to establish reliable criteria for identifying human FST, thereby clarifying its role within the motion-processing system.

## 2 Methods

### 2.1 Observers

Nine observers (four males, age 18 to 50 years) with normal or corrected-to-normal vision participated and provided written informed consent. All observers scored five or higher (70 s of arc or better) on the Randot Circles Stereotest (Stereo Optical Company, Chicago, IL), with a mean score of 8.7 and a standard deviation of 1.3. All observers participated in one scanning session for the functional localizers and two additional sessions for the population receptive field mapping. Each scanning session lasted 1.5 h. The experiments were approved by the University Committee on Activities Involving Human Subjects at New York University Abu Dhabi.

### 2.2 Apparatus, display, and MRI data acquisition

We generated stimuli on a Macintosh computer using MATLAB 9.2 (The MathWorks, Natick, MA, USA) and the Psychophysics Toolbox extensions (Brainard, 1997;Kleiner et al., 2007;Pelli, 1997). Stimuli were presented using a ProPixx DLP LED projector (VPixx Technologies Inc., Saint-Bruno-de-Montarville, QC, Canada; refresh rate: 120 Hz, screen resolution: 1920 × 1080 pixels) with a rear-projection screen (viewing distance: 88 cm; projected screen width: 38.5 cm) positioned at the back of the scanner. The display luminance was 107 cd/m² with a linearized lookup table. Stereoscopic presentation was achieved using a DepthQ Polarization Modulator from VPixx Technologies, placed in front of the ProPixx projector. Circular Polarizers from Edmund Optics were used as lenses, held in place by an MRIFocus lens holder from Cambridge Research Systems, mounted on a 64-channel head coil.

MRI data were acquired on a Siemens Prisma 3T full-body MRI scanner (Siemens Medical Solutions, Erlangen, Germany) using a 64-channel head coil. For each observer, a T1-weighted anatomical scan was acquired (TR: 2400 ms; TE: 2.22; flip angle: 8°; 0.8 mm isotropic voxels). This anatomical volume was used for white/gray matter segmentation and co-registration with the functional scans. T2^˚^-weighted functional scans were acquired using an echo-planar imaging (EPI) sequence (TR: 1000 ms; TE: 37 ms; flip angle: 68°, multiband factor: 6; matrix size: 104 × 104, 2 mm isotropic voxels; 72 slices). We also collected MP2RAGE sequences to obtain additional T1-weighted images (TR: 5000 ms; TE: 2.98 ms; TI1: 700 ms; TI2: 2500 ms; flip angle 1: 4°; flip angle 2: 5°; 176 slices per slab; 1 mm thickness; echo spacing: 7.14 ms; slice partial Fourier: off).

### 2.3 2D-motion localizer

**Fig. 1.**
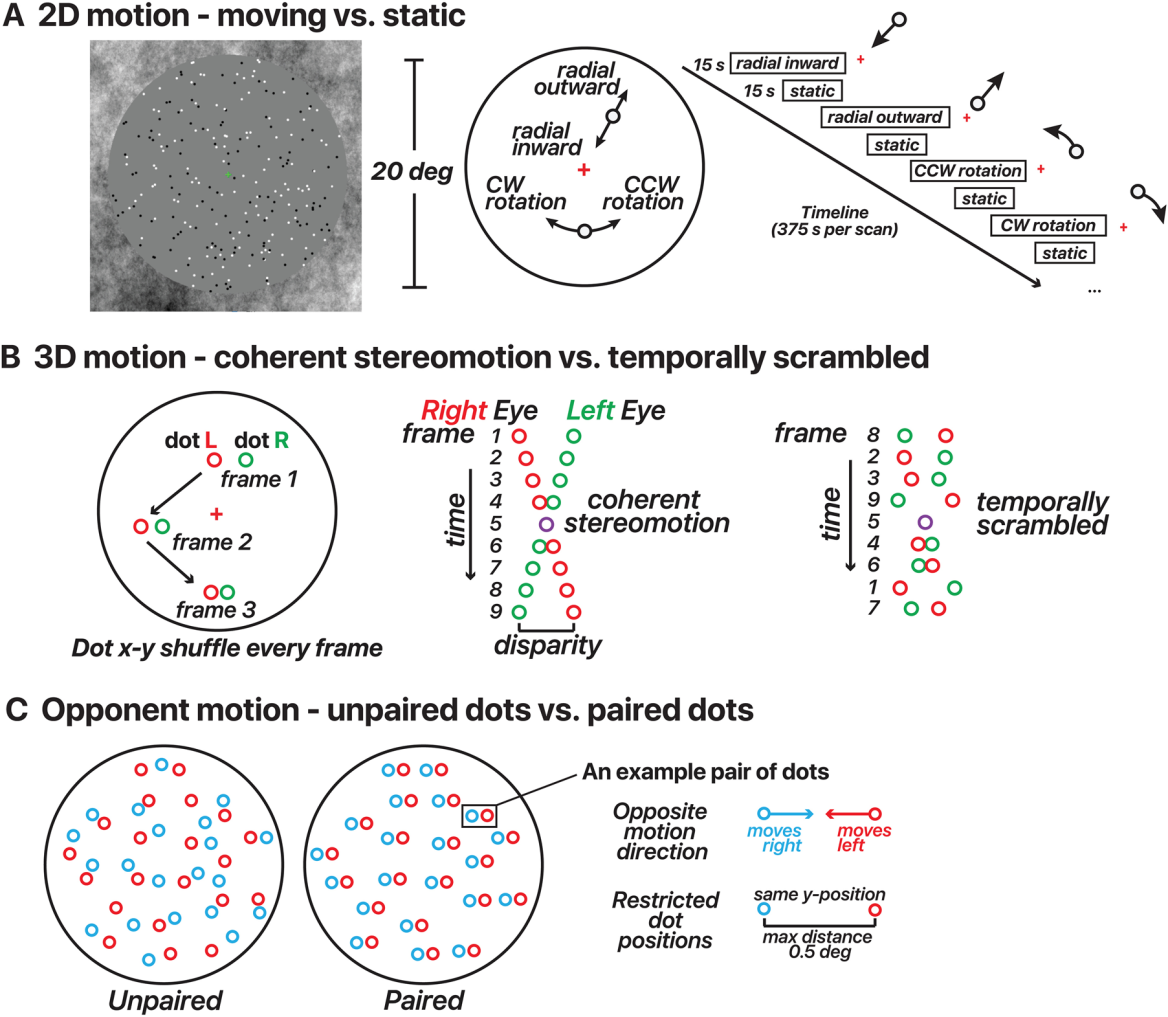
Motion stimulus design for 2D/3D-motion localizers and opponent motion. (A) 2D-motion localizer. Dots alternated from moving (inward, outward, clockwise, or counterclockwise) to static in 15 s blocks across the run. A fixation cross was located at the screen center. (B) 3D-motion localizer stimulus design (changing-disparity-defined stereomotion). Dots were binocularly presented. Red dots depict those presented to the right eye. Green dots depict those presented to the left eye. Red/green colors in the figure are for illustration. Dots were displayed in blocks as either coherent stereomotion (perceived as 3D motion toward (1 s)—away (1 s) in a central disk and away (1 s)—toward (1 s) in a surrounding annulus) or temporally non-coherent (scrambled) motion in 10 s blocks across the run. In both conditions, the dots’*x*-*y*coordinates were shuffled every stereo frame pair; the only difference was whether the disparity changed coherently or randomly (temporally scrambled order of stereo frame pairs). All dot pairs moved together in both conditions. (C) Opponent-motion stimulus design. Dots were displayed in blocks as either unpaired (15 s) or paired (15 s). In both conditions, half of the dots moved rightward and half the dots moved leftward. For illustration, the dots were color coded in this panel by motion direction (red/blue); note that in the actual experiment all dots were white regardless of the motion direction and condition. In the unpaired condition, the dots’ positions were random. In the paired condition, the two dots within a given pair had the same*y*-coordinates and were never more than 0.5 deg apart.

The 2D-motion localizer consisted of 250 black and white dots presented within a 10 deg radius circular aperture (Fig. 1A;Supplementary Video S1). Each dot was 0.2 deg in diameter and was presented for a limited lifetime of 0.5 s before reappearing in a random location. The background within the aperture was gray, and the area outside the aperture contained 1/*f*noise to aid fixation and vergence. The stimuli were presented using a block design with dots either static or moving (motion directions: radial inward, radial outward, counterclockwise rotation, and clockwise rotation). In blocks containing moving dots, the velocity of each dot depended on the eccentricity. For radial motion, the dot velocity increased as a function of the square root of eccentricity (maximum possible speed of 12 deg/s). For the rotational motion, we used one-eighth power scaling, rather than customary square-root scaling to ensure reasonable movement across the stimulus aperture—square-root scaling resulted in imperceptibly slow motion near the fovea. All four motion conditions had the same maximum speed of 12 deg/s. Each motion direction was repeated 3 times, lasting 6 min per run, and ended with 15 s of blank screen. Participants performed a color change-detection task to ensure fixation throughout each scan. A minimum of two scans were collected per participant.

### 2.4 3D-motion localizer

To attempt to delineate FST from other nearby motion-sensitive areas, we designed a stimulus that contained coherent 3D, but not 2D, motion signals (Fig. 1B;Supplementary Video S2). A similar stimulus design was used previously byLikova and Tyler (2007). We presented 100 black and white dots within a gray aperture and 1/*f*background. The stimulus consisted of dynamic random-dot stereograms. When a participant fused the images received by each eye, this resulted in a perceived depth for each fused dot that depended on the binocular disparity. To strengthen the 3D percept, we created two disparity-defined non-overlapping surfaces (a circle and a surrounding annulus). Dots within the central 6 deg of eccentricity always moved in the opposite direction in depth as dots beyond that eccentricity. The stimuli included either coherent stereomotion (3D-motion condition) or temporally scrambled disparities (control condition), which were displayed in a blocked design. In the coherent stereomotion condition, the two disparity-defined surfaces started at the near and far sides of the volume (±18 arc min disparity), and were perceived as moving in opposite directions, toward and away from the observer, both reversing direction every 1 s. The perceived 3D motion in this condition depended on the temporal (framewise) coherence of the disparity changes across all stereo dot pairs. The temporally scrambled condition (control) had temporally shuffled frames, retaining the range of binocular disparities presented (i.e., containing the same static depth information). Each possible relative disparity between the two disparity-defined surfaces was presented every second in both the coherent-stereomotion and scrambled conditions. The two surfaces remained discernible for each stereo frame pair, but the coherent 3D stereomotion percept was eliminated across frames.

Critically, the coherent-stereomotion stimulus contained 3D-motion signals (perceived as toward/away) in the absence of (spatially coherent) 2D motion signals. Thus, the two conditions were indistinguishable based on the monocular images; when closing either eye, the participant would perceive dots moving randomly in the image plane. Stereo dot pairs had the same luminance. The stimulus alternated between the coherent-stereomotion and temporally scrambled condition every 10 s. Participants reported changes of the shape of the central fixation marker (o vs. +) to ensure fixation throughout the scan. Each scan contained 5 min of stimulus presentation (15 repetitions of each condition) followed by 15 s of blank screen at the end.

### 2.5 Opponent motion stimuli

We adapted stimuli used previously byQian and Andersen (1994)to identify cortical areas with reduced responses to opponent motion (Fig. 1C;Supplementary Video S3). In each scan, we alternated a paired-dots and an unpaired-dots condition (Fig. 1C). In both conditions, 150 dots moved leftward and 150 dots moved rightward (constant speed of 5 deg/s). Each condition always consisted of 300 white dots per frame. In the paired-dots condition, the distance between paired dots could reach a maximum of 0.5 deg (resulting in a lifetime of 0.1 s). The dots within a pair had the same*y*-positions and moved one time across each other along the*x*-coordinate during their given lifetime. For the unpaired-dots condition, the*y*-positions were random. The stimulus alternated between paired and unpaired conditions in 15 s blocks across the scan, and the entire scan lasted 5 m and 15 s.

### 2.6 Receptive field mapping

We conducted experiments to map population receptive fields (pRFs) in putative FST and compared them to other motion-selective regions with topographic organization. We collected data for retinotopy using a stimulus and procedure previously described in detail (Benson et al., 2018). The stimulus content included colorful objects displayed on a 1/*f*noise background. The stimulus was confined within an aperture mask that changed position throughout the run. The procedure used two different types of apertures. Data using each type of aperture were collected as separate scans. The apertures in the first scan were slowly rotating (clockwise/counterclockwise) wedges and expanding/contracting rings. The second type of aperture was a translating bar. To better drive motion-sensitive areas, additional runs were collected with an adapted version displaying moving stimuli behind the same apertures. The motion was created by expanding and contracting the original images in 0.6 s cycles. Each scan lasted 5 min, and 12 scans were collected in total: 6 scans that were the same as used byBenson et al. (2018)and 6 scans of the adapted moving version. For these scans, the stimulus-aperture radius was increased to 12.2 deg.

### 2.7 Pre-processing and statistical analysis

All scans were organized using the Brain Imaging Data Structure (BIDS) format (Gorgolewski et al., 2017). We then used the fMRIPrep pipeline (version 20.2.3) for motion correction, spatial normalization, and co-registration of functional and anatomical scans (Esteban et al., 2019). This step also converted our data into the fsaverage and fsnative spaces, which represent the data on the surface of the cortex with units of vertices instead of voxels. All subsequent analyses were conducted on the surface data.

We ran a separate general linear model for each localizer analysis. The regressors included boxcar functions representing the full stimulus duration, convolved with a canonical hemodynamic response function (hRF), a constant, a linear drift, and six translational and rotational motion regressors derived from fMRIprep. Before regression, the fMRI data were converted to percent signal change by normalizing each voxel’s time series to its mean. The beta estimates were computed using the pseudoinverse of the design matrix. The beta weights were estimated separately for each run and subsequently averaged.

To derive the response to 2D motion, we subtracted the beta weights for static stimuli from the beta weights for moving stimuli. Throughout the remainder of this paper, we refer to the response to 2D motion as this contrast. Similarly, to derive the response to 3D motion, we subtracted the beta weights for the temporally scrambled condition from the beta weights for the coherent-stereomotion condition. For motion opponency, we subtracted the beta weights for the paired-dots condition from the beta weights for the unpaired-dots condition. We defined the strength of motion opponency as the degree of decrease in response between the unpaired- and paired-dots conditions. The above analyses resulted in one beta weight per vertex per metric (2D, 3D, opponency).

To estimate the cortical myelination pattern, we merged the MP2RAGE scans to create a uniform T1-weighted image (UNI), from which T1 maps were estimated using qMRLab (Karakuzu et al., 2020). The longitudinal relaxation rate (R1) was calculated as R1 = 1/T1, which has been shown to correlate with myelin content (Mancini et al., 2020). This myelin density estimate provided an additional structural metric to evaluate the distinctiveness of functionally-localized regions, particularly in distinguishing putative FST from hMT/MST.

We processed the pRF data by first averaging all bar runs and all wedge/ring runs. Then using*Vistasoft*(https://github.com/vistalab/vistasoft), we derived estimates of each vertex’s preferred visual field position (*x*,*y*) and size (*σ*). This procedure modeled each pRF as a 2D circular Gaussian using a coarse-to-fine, multi-stage optimization procedure previously described in detail (Dumoulin & Wandell 2008;Himmelberg et al., 2022). The*x*,*y*position was then transformed to polar coordinates to represent eccentricity (*ρ*) and polar angle (*θ*).

Functionally-defined regions of interest (ROIs) were drawn and evaluated separately within each native hemisphere (seeSection 3.1for details on ROI definition). For group-level comparisons, such as evaluating whether myelin density is greater in hMT/MST compared to pFST, the values were always derived from each subject’s native hemispheres. The only instance where data were transformed out of native space was insection 3.6, when we assessed the alignment between atlas-defined and functionally-defined ROIs by transforming individual subject ROIs into Freesurfer’s*fsaverage*space to compute their average group position. Throughout the study, any reference to “atlas-based” ROIs corresponds to those derived from the Glasser atlas (Glasser et al., 2016).

## 3 Results

### 3.1 Criteria for localizing putative FST and hMT/MST

In our analyses, we first used the results from the 2D- and 3D-motion localizer stimuli to delineate hMT/MST from pFST. We then assessed the plausibility of pFST being functionally distinct from hMT/MST by assessing the differential activation of pFST to opponent motion, distinct population receptive field properties, and estimates of myelin density.

Traditionally, functional localizers are used to elicit activity from a particular cortical region whose function is distinct from neighboring regions. For example, it is common to select voxels that have greater responses to moving than static dots to isolate the MT complex, which contains several motion-selective areas (DeYoe et al., 1996). However, a single functional localizer may activate multiple cortical areas (e.g., voxels in the primary visual cortex, in addition to the MT complex, may respond more to moving than static dots). Conversely, a single cortical area may respond to various functional localizers or visual features (e.g., V1 shows selectivity for both orientation and motion). Our study adheres to assumptions rooted in prior literature (mainly studies performed with macaque monkeys) to localize pFST. The existence of non-visual and non-motion-responsive cells within FST (Erickson et al., 1989) complicates the interpretation of fMRI response amplitudes to motion stimuli and it is not clear if human FST is activated by 2D-motion localizers. Consequently, we posited the following assumptions to guide our delineation.

The 2D-motion localizer (Fig. 1A) was expected to consistently activate hMT/MST, with the potential to also engage pFST. This assumption was informed by the established response of all three areas to 2D-motion stimuli, albeit with a predisposition for hMT/MST activation. We anticipated that the peak activation elicited by the 2D-motion localizer would lie within hMT/MST rather than pFST, reflecting the primary association of 2D-motion processing with the former regions. Conversely, the 3D-motion localizer (Fig. 1B) was likely to activate pFST, given its role in processing 3D motion (Thompson et al., 2023). Activation of hMT/MST by this localizer was possible but not guaranteed given the heterogeneous findings about 3D-motion processing in macaque MT (Czuba et al., 2014;Sanada & DeAngelis, 2014;Thompson et al., 2023). Thus, we left open the possibility that the peak activation for 3D motion may occur in either region of interest (ROI).

These assumptions underpinned our approach to delineate ROIs as illustrated inFigure 2Afor which three possible activation patterns are highlighted. The first scenario, which we observed in some instances, involved non-overlapping activations where 2D- and 3D-motion localizers elicited robust activation from distinct but neighboring areas (example shown inFig. 2B). The second scenario, which we observed in the majority of hemispheres, included cases where the 3D-motion localizer activated a larger area compared to the 2D-motion localizer. In these cases, the 2D-motion localizer activated a subset of the 3D-activated region (example shown inFig. 2C). The third scenario, which we did not observe in the data, were potential cases in which the 3D-motion localizer activated a subset of the 2D-motion localizer. Following the above logic, the area activated by 2D and possibly 3D motion was identified as hMT/MST whereas the remaining nearby area activated by 3D but not 2D motion was identified as pFST.

**Fig. 2.**
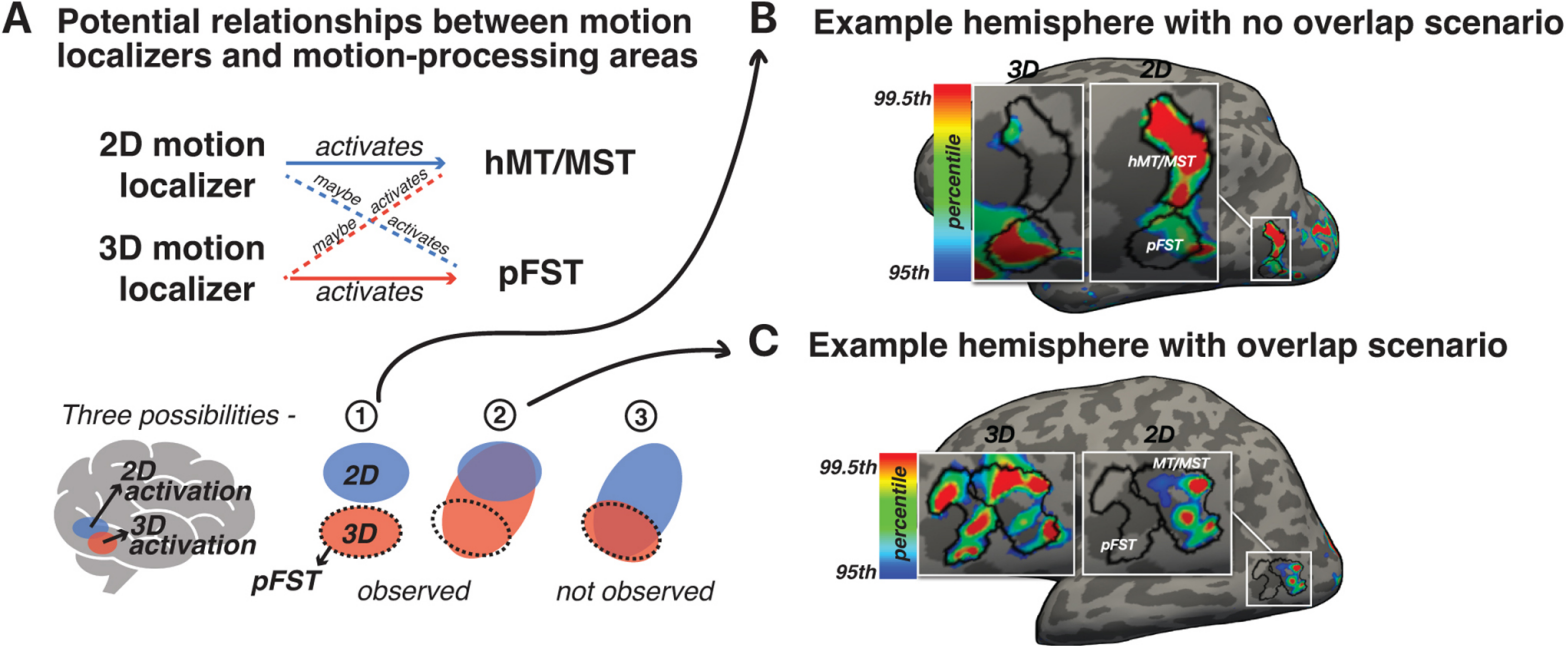
Delineating pFST. (A) Potential relationships between motion localizers and motion-processing areas. Drawing of pFST is depicted under three different scenarios based on the activation of 2D- and 3D-motion localizers. Blue patches represent selectivity to 2D motion, and red patches represent selectivity to 3D motion. The dashed circle marks the potential drawing of area pFST. The three scenarios include (1) no overlap, (2) partial overlap where the 2D-motion localizer activates a subset of the 3D-motion activation, and (3) partial overlap in the reverse direction. The third scenario was not observed in the data. (B) Example hemisphere with a no-overlap scenario. Activation maps are shown on an inflated surface, thresholded at the 95th percentile. (C) Example hemisphere with an overlap scenario. In this case, the 2D-motion localizer activated a subset of the 3D motion activation.

To manually draw hMT/MST and FST, we displayed the contrast maps for the 2D- and 3D-motion localizers on the cortical surface using Freesurfer’s Freeview tool. To prepare for delineation, we set a threshold for the 2D- and 3D-motion localizer responses to display vertices that were ≥95th percentile across the entire hemisphere. To better evaluate the separation of the 2D and 3D activation patterns, we then dynamically adjusted the threshold toward the 90th percentile, with no thresholds set below the 90th percentile. Adjusting individual thresholds in this way accommodates inter-subject variability in activation magnitude and spatial distribution (Fox et al., 2009;Gorgolewski et al., 2012;Stevens et al., 2013). Functional localizers reliably identify regions of interest. However, the ROI boundary will vary with threshold and we chose to draw ROIs conservatively. This may have led us to underestimate their size, but it increased the specificity of the vertices labeled as hMT/MST or pFST. Similarly,Huk et al. (2002)adjusted statistical thresholds to clarify boundaries between hMT+ and neighboring patches of activation, excluding adjacent activity when higher thresholds revealed clearer separations. For comparison, both the 2D- and 3D-motion activations are shown for every hemisphere at both the 95th and 90th percentile with ROI outlines inSupplementary Figure S1. Importantly, the value of the threshold did not affect the activation-pattern scenarios (e.g., whether 2D and 3D activations were overlapping or non-overlapping).

We defined hMT/MST as the region activated by the 2D-motion localizer and drew it first because (1) this localizer is widely used as the operational defining criterion for hMT+, and (2) the 3D-motion localizer activation sometimes overlapped with parts of hMT/MST. To aid in manually segmenting pFST, we discerned which parts of the cortex were highly responsive to the 3D-motion localizer and not within hMT/MST. To do so, we also used a position criterion to draw pFST—it was generally located ventral and/or anterior and adjacent to hMT/MST. This criterion was crucial in cases where the 3D-motion localizer resulted in multiple peak activations. We only considered vertices located within or near the lateral-occipital and inferior temporal gyri, and assumed contiguous vertices within hMT/MST as well as FST. For a detailed, step-by-step guide on how we drew the ROIs, see:https://github.com/raniaezzo/DrawingROIs.

### 3.2 Location of pFST across individual hemispheres

**Fig. 3.**
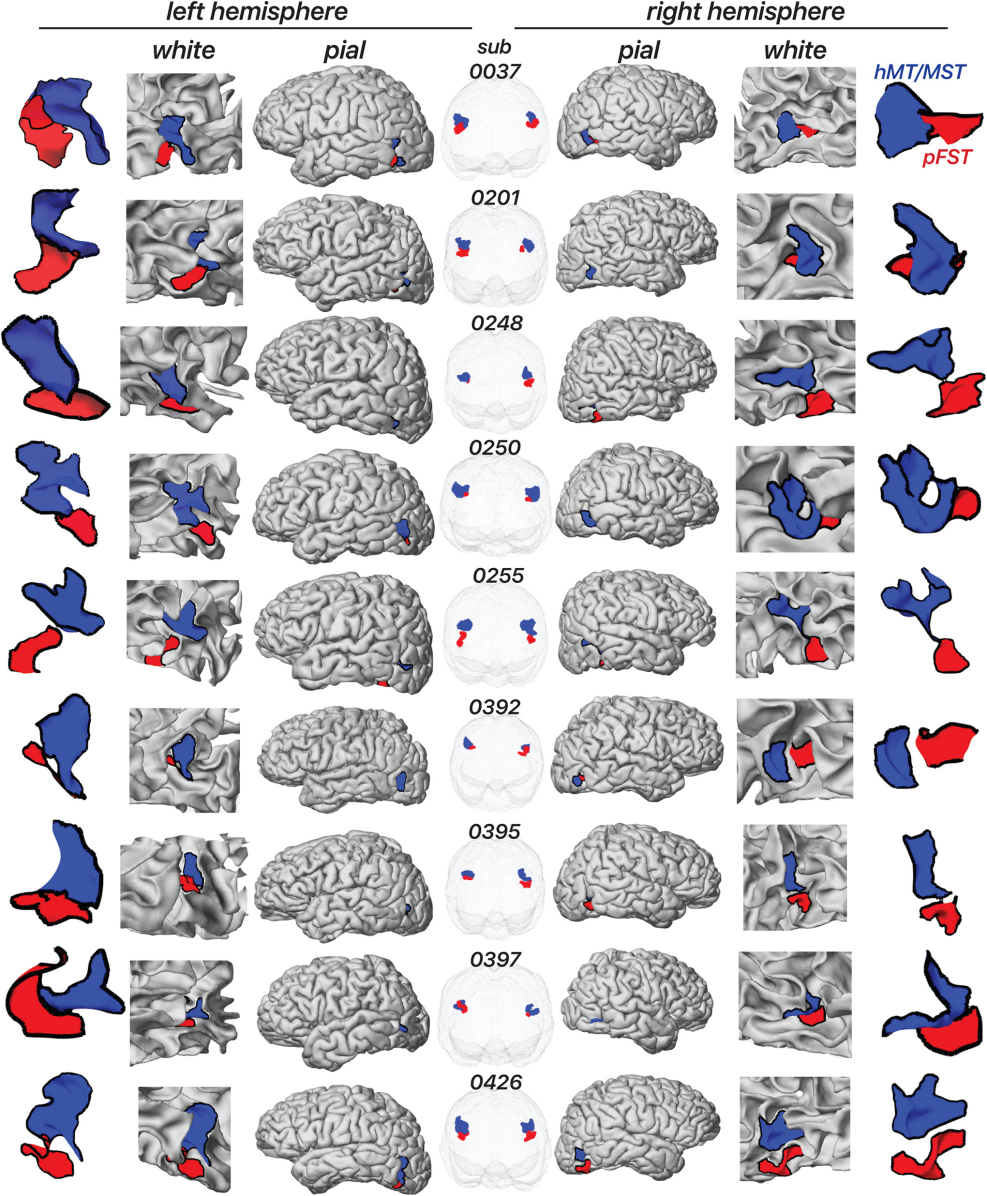
Location of area pFST and hMT/MST across individual hemispheres on the white and pial surface as well as in a glass-brain view. Each row shows a separate participant. The blue area represents hMT/MST, and the red area represents pFST.

Figure 3illustrates the location of pFST (red) in comparison to hMT/MST (blue) across individual hemispheres, presented on both white and pial surfaces as well as in a glass-brain view. We consistently localized pFST anterior and/or inferior to hMT/MST, a pattern that remains relatively consistent across individuals when viewed in the volume. Anatomically, and consistent with prior work (Dumoulin et al., 2000), both pFST and hMT/MST were in the vicinity of the ascending limb of the inferior temporal sulcus (ITS), near a sulcus that runs almost perpendicular to the ITS/STS, between the temporal and occipital lobes. While consistent in relative location to hMT/MST, the shape and size of pFST exhibited considerable variability between individuals, which was more noticeable when projected onto cortical surfaces. The largely symmetrical positioning of pFST across left and right hemispheres within individuals suggests some consistency in the neuroanatomical organization of motion processing.

### 3.3 Evaluating complementary measures in a representative hemisphere

In this section, we illustrate each of the analyses performed using an example hemisphere. We expand on these analyses inSection 3.5, which presents the results for all subjects at the group level. This organization is for ease of interpretation: each manually drawn ROI is unique in size, shape, and position, and our validation criteria were evaluated on a hemisphere basis. The complementary measures used to validate the regions defined as hMT/MST and pFST include opponent motion and myelination.

First, to establish whether the identified regions were motion selective, we tested whether regions hMT/MST and pFST were more activated by motion conditions for both the 2D- and 3D-motion localizers. In the example hemisphere inFigure 4, hMT/MST responded more strongly in the moving versus the static condition (*t*(295) = 32.49,*p*< 0.0001), as well as in the 3D stereomotion versus temporally scrambled control condition (*t*(295) = 16.42,*p*< 0.0001). Similarly, pFST also responded significantly more strongly in the 2D (*t*(94) = 13.42,*p*< 0.0001) compared to static, and in the 3D-motion condition (*t*(94) = 25.53,*p*< 0.0001) compared to the temporally scrambled control.

**Fig. 4.**
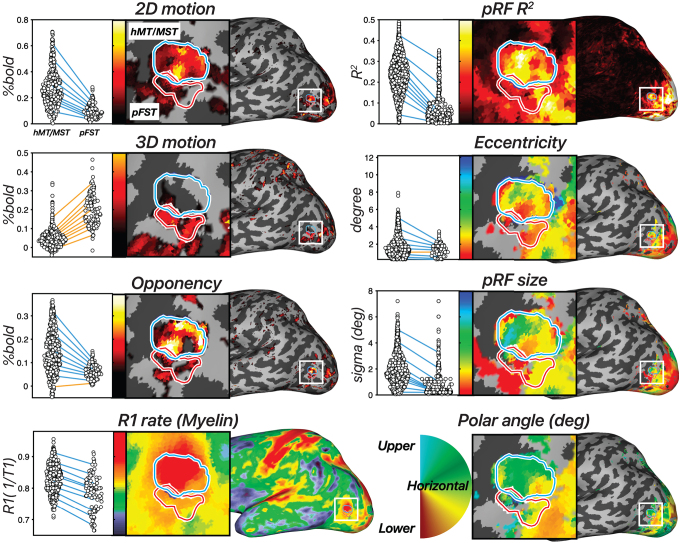
Results across 8 measures in an example hemisphere. hMT/MST is labeled with a blue outline, and pFST is labeled with a red outline. [left, first row] 2D-motion response (2D moving—static dots), [left, second row] 3D-motion response (coherent stereomotion—temporally scrambled dots with disparity), and [left, third row] motion opponency (unpaired—paired moving dots) thresholded at the 90^th^percentile. [left, fourth row] R1 rate (1/T1). Higher values are associated with greater myelin density. In the dot plots, each dot represents a single vertex from the surface and each line connects each of the 10^th^percentiles of the distribution across the two ROIs. Negative slopes (hMT/MST > pFST) are colored blue, and positive slopes are colored orange [right, first to fourth rows]. Estimated pRF parameters: variance explained (*R*^2^), eccentricity (deg), pRF size (deg), and polar angle (angle 0–360). All pRF results are thresholded at*R*^2^> 10%.

Second, we compared responses between hMT/MST and pFST across localizers, as a means to validate their unique functional and structural characteristics. As expected, hMT/MST responded significantly stronger to 2D than to 3D motion (*t*(295) = 24.55,*p*< 0.0001). For pFST, the responses to 2D and 3D motion were not significantly different (*t*(94) = -1.7147,*p*= 0.0888).

We then used motion opponency as a complementary measure to verify the boundaries. The aim was to discern whether the region identified as pFST is, indeed, a separate area or simply an extension of the well-characterized hMT/MST area. In the example hemisphere, hMT/MST had significantly stronger signal suppression to opponent motion (Fig. 1C) compared to pFST (*t*(389) = 8.29,*p*< 0.0001). This fits with the established understanding that areas like hMT, similar to their macaque counterparts, demonstrate a marked decrease in activity when presented with opponent-motion stimuli.

We also examined cortical myelination patterns using R1 rate, the inverse of the T1 relaxation time, as a proxy. We anticipated greater myelination in hMT/MST than pFST based on the literature (Abdollahi et al., 2014;Glasser et al., 2016;Krubitzer & Kaas, 1990;Rosa et al., 1993). Average T1 relaxation time for gray matter in the human brain is about 1.331 s (Wansapura et al., 1999), which corresponds to a 0.751 s^−1^R1 rate. Consistent with this, the median R1 rate across the whole hemisphere was 0.749 s^−1^for this participant. The R1 rate for hMT/MST was 0.838 s^−1^and for pFST was 0.792 s^−1^with significantly greater values in hMT/MST than pFST (*t*(389) = 7.69,*p*< 0.0001), suggesting greater myelination in hMT/MST. However, R1 rates in both hMT/MST and FST were significantly greater than the average cortical R1 rate (*t*_hMT/MST_(295) = 26.57,*p*< 0.0001;*t*_pFST_(94) = 4.76,*p*< 0.0001), suggesting that myelination in these motion-related areas may be greater than other cortical areas. This myelination contrast served not as a localizing tool but as a confirmatory measure to validate the distinction between pFST and hMT/MST.

Additionally, 12 runs of the pRF-mapping stimulus were collected for each subject to compare the receptive-field properties between regions. In the literature, two key points have been relied upon in isolating FST in non-human primates. First was the presence of significantly larger receptive field sizes in FST compared to other areas in the MT complex. Second, polar angle estimates represent the vertical meridian at the borders of FST with MST and V4t. There is a debate in the literature about the extent of retinotopic organization in FST. Some reported large receptive fields prioritizing central vision with no clear retinotopic organization in macaques (Boussaoud et al., 1990;Desimone & Ungerleider, 1986;Erickson et al., 1989), galagos (Krubitzer & Kaas, 1990), and Cebus apella monkeys (Rosa et al., 1993). Others identified a complete visual-field representation of FST in macaques, including the lower vertical meridian separation from MST and the upper vertical meridian separation from V4t (Kolster et al., 2009).

However, our pRF results did not ultimately prove useful in delineating pFST. Area pFST, in particular, showed low*R*^2^values (median*R*^2^= 11.17%), which compared unfavorably to hMT/MST (median*R*^2^= 23.19%). This may be due to several factors. One possibility is that pFST has either extremely coarse or a complete lack of retinotopic organization. Another possibility is that pFST is retinotopically organized but cannot be topographically mapped using conventional methods due to constraints imposed by the limited field of view for visual stimulation within the scanner. Nevertheless, a lack of clear topology in pFST was a consistent observation across participants. We consequently ran several simulations to understand potential reasons for these outcomes.

### 3.4 Retinotopic mapping differentiates hMT/MST and pFST

Retinotopic mapping, and in particular population receptive-field mapping, can be used to localize and delineate areas along the visual processing hierarchy, including hMT and MST (Amano et al., 2009;Huk et al., 2002;Kolster et al., 2010). However, this approach relies on the ability to reliably distinguish the receptive fields of different neural populations in response to localized stimuli across the visual field.

In monkey FST, receptive fields are substantially larger than in MT, with a radius that is larger by a factor of ~1.5–2.2 (Thompson et al., 2023). The visual field available for stimulation in a human MRI experiment is limited by the bore size of the scanner, typically limiting the field of view to the central 15–30 deg. In the experiments conducted here, stimuli were restricted to the central ~24 deg. Practically speaking, this meant the receptive-field size in pFST in humans would likely approach, or exceed the size of the available field of view, as defined by the stimulus aperture.

To empirically probe the efficacy of pRF mapping in pFST, we conducted retinotopic-mapping experiments in all of our observers. We subsequently fit two models to the data, a standard pRF model and a simple stimulus-contrast model. For the first model (pRF model), the responses depended on the visual field position of the bar stimulus relative to the pRF (peaking at timepoints when the stimulus maximally overlaps the pRF). For the second (stimulus-contrast) model, pRF responses were not specific to the stimulus position, but instead based on the total area of stimulus content, corresponding to the size of the aperture present on the display (peaking at timepoints when the stimulus/aperture covered the largest amount of the visual field).

**Fig. 5.**
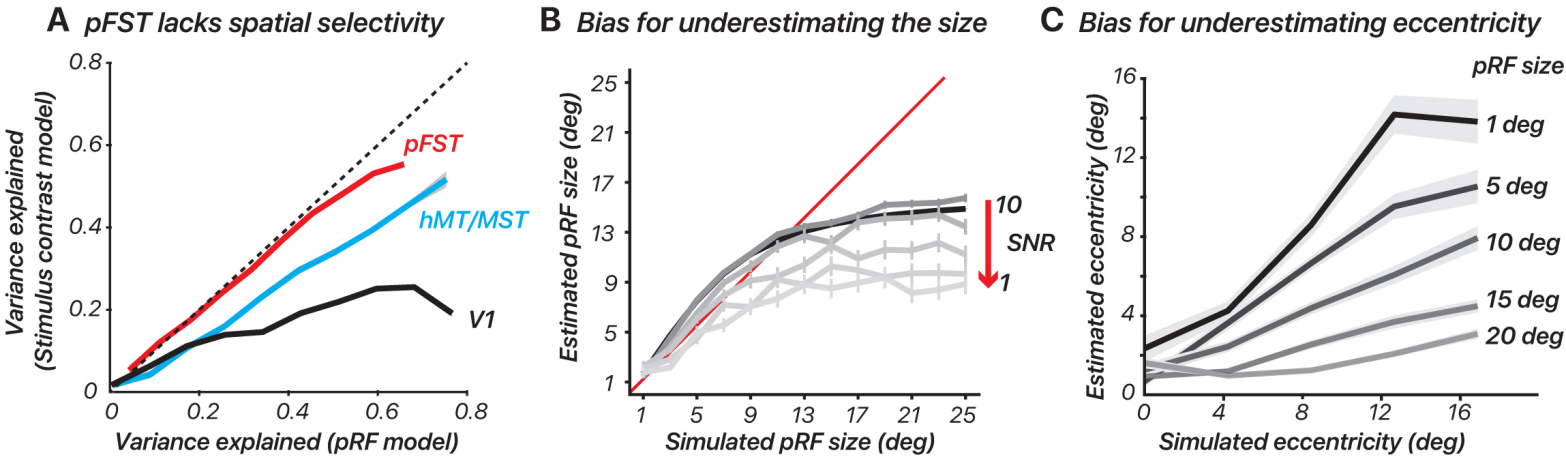
Retinotopic mapping differentiates hMT/MST and pFST. (A) Variance explained for one subject, using the pRF model versus stimulus contrast (ON/OFF) model in V1, hMT/MST, and pFST. (B, C) pRF size and eccentricity were underestimated for large simulated pRFS, especially with low signal-to-noise ratio (SNR). Error bars represent the standard error across bootstrap simulations.

We calculated the variance explained for each surface vertex using both models in V1, hMT/MST, and pFST (Fig. 5A). In V1, and to a lesser extent hMT/MST, variance explained was substantially greater for the pRF model than the stimulus-contrast model, suggesting that the spatial specificity of the neural population could be resolved within the stimulus aperture (diameter = 24.4 deg). In pFST on the other hand, variance explained was not substantially different between the two models. This suggested either that neurons in pFST are not retinotopically organized, or that the size of receptive fields within pFST approached or exceeded our stimulus aperture and therefore could not be estimated.

Given that prior work proposed a pFST region with coarse topographic organization using fMRI (Kolster et al., 2010), we investigated which factors could lead to a lack of clear topographic organization. To accomplish this, we assessed how noise levels influence estimated pRF size and eccentricity for areas with large receptive fields. We simulated BOLD time series for one of our retinotopic-mapping stimuli (the sweeping bar) and defined parameters for simulated pRF including eccentricity, polar angle, and size.

We simulated responses for these two models by iteratively increasing the receptive-field size and noise level. We expected these two factors to systematically result in poorer pRF estimates. Noise was defined as the standard deviation of Gaussian noise added to the modeled fMRI signals. By adjusting the noise standard deviation, the SNR was manipulated, simulating conditions ranging from low to high noise.

Consistent with our retinotopy estimates for pFST, the pRF simulations showed an underestimation of pRF size. This underestimation was more pronounced as noise levels increased (Fig. 5B). We also evaluated how simulated pRF size influenced eccentricity estimates. We simulated pRFs with various sizes (1, 5, 10, 15, 20 degrees of visual angle) and center locations (0, 3, 6, 9, 12 degrees from the visual field center) for a group of 50 voxels. The fMRI response to each visual stimulus was calculated by convolving the stimulus profile with the hRF and adding noise. The pRF model was then applied to estimate the size and location of the pRF from the simulated fMRI data.

The results showed that estimating pRF eccentricity is inherently affected by the pRF size, with larger sizes significantly increasing the bias towards underestimating the eccentricity (Fig. 5C). Taken together, these results confirm that retinotopic mapping is poorly suited to localizing and delineating visual areas whose receptive fields approach or exceed the size of the stimulus that can be presented. In our simulations, this led to results in which both pRF size and eccentricity were consistently underestimated. In cortical areas for which such concerns arise, simultaneously testing a simple stimulus-contrast model will be useful. If the variance explained does not improve meaningfully with a pRF model, this suggests that estimates of pRF size and eccentricity will be biased.

### 3.5 Triangulating human pFST: Group-level validation with motion opponency and myelination

**Fig. 6.**
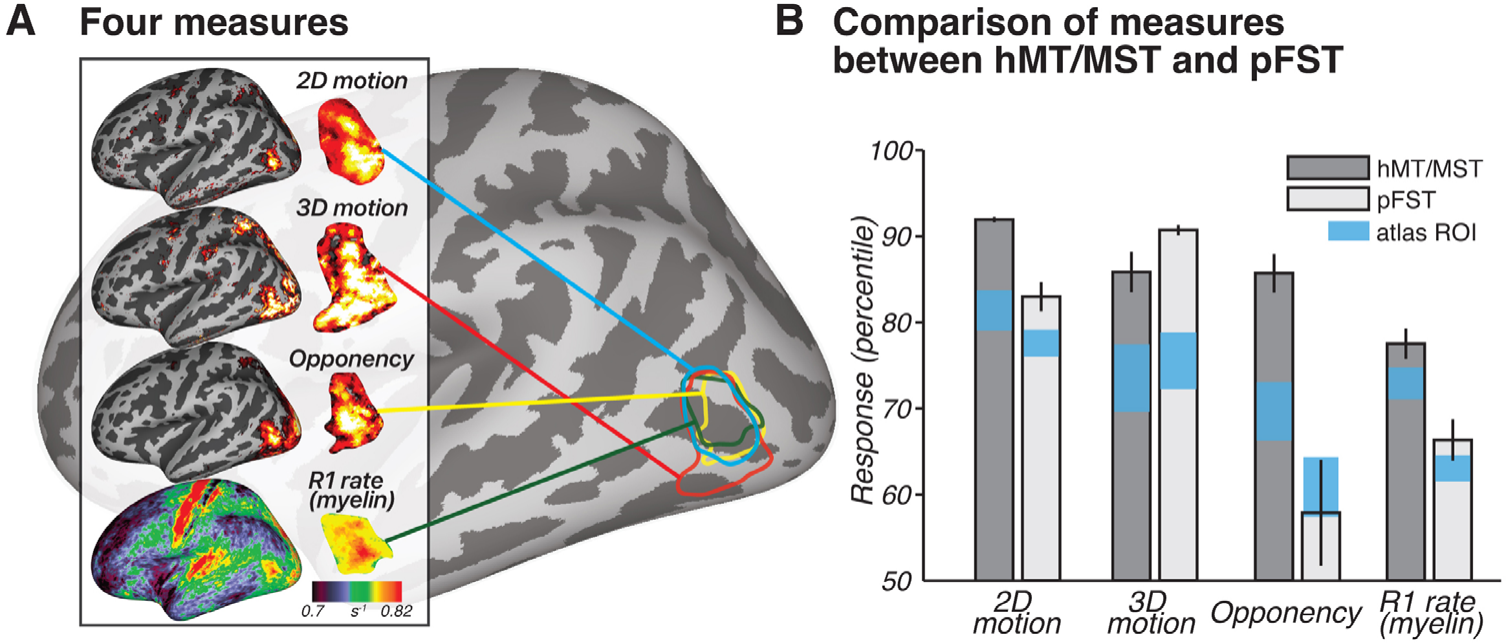
Group-level results. (A) Four measures across subjects (*n*= 9) to 2D motion (moving–static), 3D motion (coherent stereomotion – temporally scrambled), opponency (unpaired- – paired-dot motion), and R1 rate (myelin) visualized on an inflated left hemisphere on the fsaverage surface. For 2D, 3D, and opponency, color scale represents the percentage change in BOLD signal, thresholded from 90^th^(red) to 99^th^percentile (yellow). For R1 rate (myelin), color scale denotes variations in myelination ranging from 0.7 to 0.82 s^-1^. (B) Comparison of these four measures between hMT/MST and pFST using the manually drawn ROIs and the Glasser atlas ROIs. Bars show mean and standard error across hemispheres (*n*= 18). Note that the values in B are plotted as percentiles for visualization purposes to emphasize the relative difference between hMT/MST and pFST. The statistical analyses reported in the text were conducted on the original values (BOLD signal change or R1 rate) without converting to percentiles. The same measure, using atlas-defined ROIs, is provided as a reference and visualized with blue squares representing SEM.

While the current pRF methods may be suboptimal for pFST, the differences in pRF results between hMT/MST and pFST support the functional distinction between these regions. This distinction is further reinforced by two additional metrics: motion opponency and myelination (Fig. 6). We identified greater motion opponency (activity for unpaired–paired motion) in hMT/MST compared to pFST across all hemispheres with a paired*t*-test (*t*(17) = 5.2307,*p*< 0.0001). In addition to the group-level results, individual one-tailed two-sample*t*-tests were carried out for each hemisphere, yielding significant results for stronger motion opponency in hMT/MST in 15 out of 18 hemispheres. The consistency of these results supports the functional distinction between hMT/MST and pFST. However, in three hemispheres (two from the same participant), there was no significant difference. This could suggest that, in a few instances, areas classified as pFST based on 3D-motion activation might partially overlap with hMT/MST.

A paired-sample*t*-test comparing myelination between hMT/MST and pFST across 18 hemispheres yielded a significant difference (*t*(17) = 5.1307,*p*< 0.0001). No significant differences in myelination were detected between hemispheres (*t*_hMT/MST_(8) = -1.5324,*p*_hMT/MST_= 0.1640;*t*_pFST_(8) = -1.4400,*p*_pFST_= 0.1878). The observed myelination patterns, in combination with the motion-opponency measures, support the conclusion that the areas we identified as pFST are functionally and structurally distinct from hMT/MST.

Interestingly, for one participant (sub-0392), both hemispheres displayed results in both the motion-opponency and myelination measures that were atypical compared to the other participants. The hemispheric consistency of the results for that participant is consistent with individual cortical variability that may have reflected several factors. First, that participant’s average hMT/MST surface area across hemispheres was 181.53 mm², slightly more than half the average surface area of hMT/MST across the other eight participants (302.77 mm²). Second, the participant reported two pre-existing medical conditions, including regular ocular migraines and small lesions in V1. Because of these differences, we verified that excluding this participant had no effect on the statistical conclusions for any of the population results.

### 3.6 Individual variability compared to atlas-based location

**Fig. 7.**
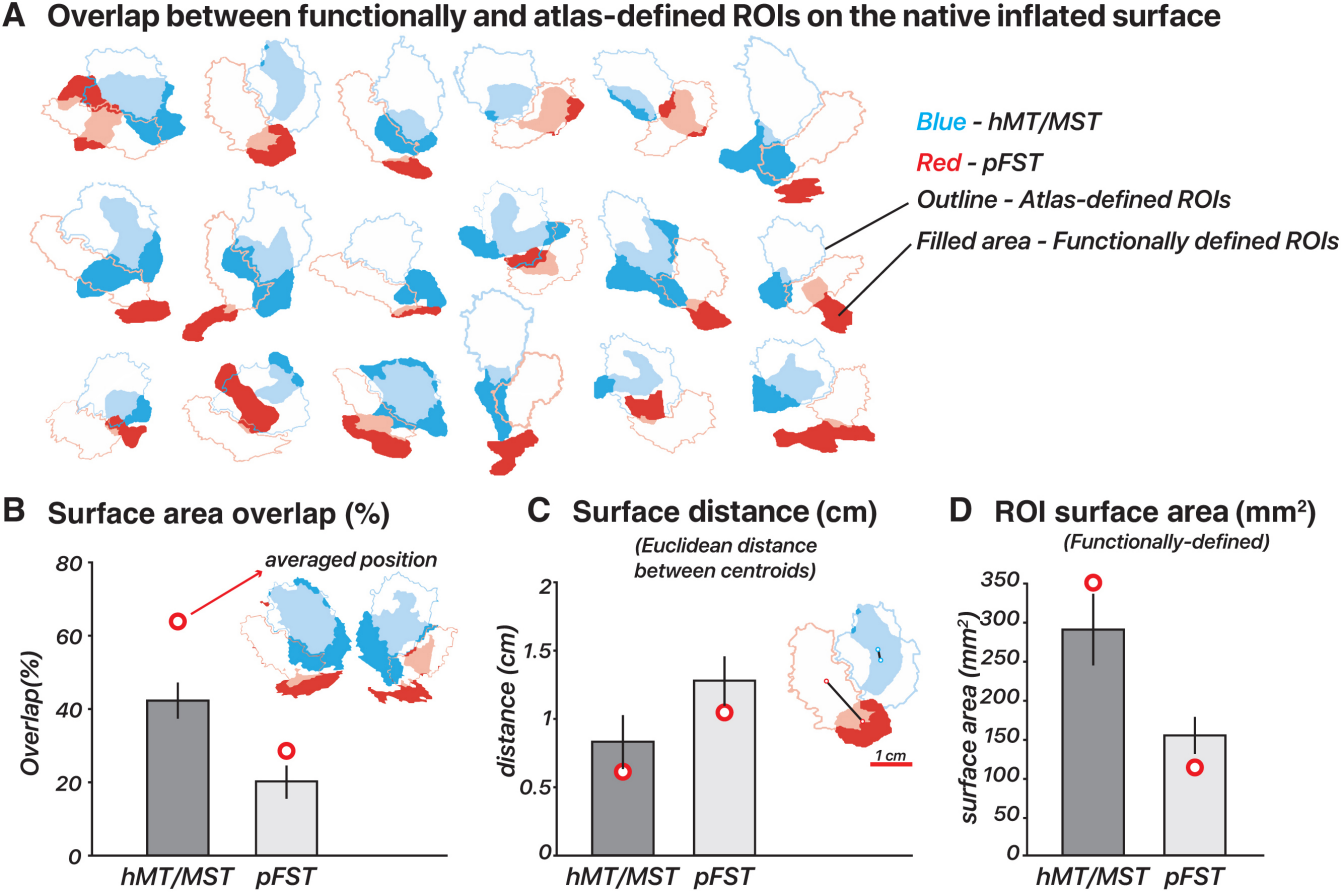
Functional versus atlas-defined pFST. (A) Functional and atlas-defined ROIs in native inflated-surface space. Each cluster represents the overlap of regions delineated based on an atlas (Glasser et al., 2016) versus functional localizer within a single hemisphere. Atlas-defined hMT and MST were combined into one ROI, which was comparable with functionally-defined hMT/MST. Color-filled areas indicate vertices from ROIs manually drawn based on our localization criteria (2D- and 3D-motion functional localizers). Atlas-defined ROIs are shown as unfilled outlines. For both functional and atlas-defined ROIs, hMT/MST is blue while pFST is red. Filled areas with semi-transparent blue/red color indicate vertices that had consistent ROI labels for atlas- and functionally-defined methods. (B) Surface area overlap (%): The bar plot shows the mean overlap percentage ± 1 standard error (black vertical line) across hemispheres in native space. Red circles mark the mean overlap value across subjects in fsaverage space. (C) Surface distance (cm): Distances in centimeters were calculated between the centroid coordinates of each ROI in inflated-surface space, with red circles marking the mean distance in fsaverage space. (D) Surface area (mm^2^): Mean surface area of functionally-defined ROIs in inflated-surface space, with red circles marking the mean area of averaged functionally-defined ROIs in fsaverage space.

We compared our functionally-defined ROIs (Fig. 7), based on responses to motion localizers, to those outlined by theGlasser et al. (2016)atlas. The Glasser atlas contains a comprehensive cortical parcellation, which is based on a combination of cortical architecture, function, connectivity, and topography. Unlike many other parcellation atlases, it contains an FST region. We quantified the agreement between the atlas-defined and functionally-defined ROIs by quantifying their spatial overlap using each delineation method. We computed the percentage overlap per ROI, in terms of the DICE coefficient, by doubling the number of overlapping vertices and dividing by the total number of vertices in both the atlas-defined and functionally-defined ROIs. The correspondence between functional and atlas-defined ROIs varied across subjects without a consistent deviation pattern. This variability was the most pronounced for pFST, with hMT/MST generally showing a greater degree of overlap and smaller centroid distances, often less than 1 cm (Fig. 7B, C). Specifically, hMT/MST exhibited a median surface overlap of 43% and an average centroid distance of 0.68 mm in native space. For pFST, the median overlap was 11% and the centroid distance was 1.36 mm in native space. Functional pFST had little overlap with atlas hMT/MST (7%). Likewise, functional hMT/MST had little overlap with atlas FST (12%). The surface area of functional hMT/MST was 289.2 mm² on average across 18 hemispheres, about twice the size of functional pFST (153.4 mm²). For comparison, the atlas-defined surface areas were both larger than the functional ROIs, with hMT/MST at 379.4 mm² and FST at 301.7 mm² (Fig. 7D).

To assess how well the atlas-defined ROIs aligned with the functionally-defined ROIs’ average position across subjects, we transformed ROIs from all subjects into fsaverage space, and each vertex of the averaged surface was labeled based on majority agreement, reflecting the average functional position across subjects. In fsaverage space, the overlap percentage increased and the distances decreased for both ROIs. Functional and atlas-defined hMT/MST had 64% overlap, with a distance of 0.60 mm. Functional and atlas-defined pFST had 28% overlap, with a distance of 1.02 mm. In both native and averaged space, we observed significant variability in the size and shape of these ROIs across individual hemispheres. On average, atlas-based methods agreed well with the average functional data (red circle inFig. 7B), but when analyzing individual hemispheres, functionally-defined hMT/MST and pFST deviated quite drastically from atlas-based parcellations (bars inFig. 7B). These deviations occurred for all subjects and could have a notable impact on statistical analyses given the relatively small number of voxels/vertices within these areas. Averaging across participants or relying solely on atlas-defined ROIs without accounting for such variability could obscure anatomical and functional differences.Figure 6Bfurther demonstrates that functionally defined ROIs (gray bars) more clearly distinguish hMR/MST from pFST across nearly all measures compared to atlas-defined (blue bars) ROIs. This consideration is likely critical for motion-selective areas of the human inferior temporal cortex, including hMT/MST and FST.

## 4 Discussion

We demonstrated that FST’s unique functional properties set it apart from neighboring motion-responsive areas located at the junction of the anterior occipital sulcus and the inferior temporal sulcus. Notably, our motion localizers revealed that pFST often responds less to 2D and more to 3D motion than other nearby areas. The known sensitivity of FST to 3D motion in macaques both motivated and provided support that this area is indeed the human homolog. An additional functional criterion—weaker motion opponency (Rosenberg et al., 2008)—validated this delineation. We further summarized anatomical and structural characteristics that can aid in identifying pFST, including its lower levels of myelin and anteroventral location relative to hMT/MST.

Our delineation of FST was largely based on functional properties in the macaque, with some assumptions regarding the general proximity to hMT/MST. In terms of the visual processing hierarchy, FST is considered to be downstream from MT/MST and to process more abstract forms of motion, with less selectivity for the direction of retinal motion than MT/MST (Desimone & Ungerleider, 1986;Saito et al., 1986). Indeed, prior work with macaques supports the hypothesis that FST processes higher-order motion, including stereomotion (Héjja-Brichard et al., 2020;Rosenberg et al., 2023;Thompson et al., 2023) and structure-from-motion (Mysore et al., 2010;Vanduffel et al., 2002). FST is also thought to process the 3D motion of objects, which is consistent with its strong connectivity to V4 and V4t, areas within the ventral pathway (Boussaoud et al., 1990). This macaque work is collectively consistent with the functional responses and anatomical location of pFST described here.

Although not directly considered an FST homolog, a cortical area selective for stereomotion was previously identified in the human brain using a similar 3D-motion stimulus (Likova & Tyler, 2007). That stereomotion region was adjacent to hMT/MST, consistent with our findings, likely activating what we identify as pFST. However, the activated cortex was on average anterior relative to MT/MST. We found similar anterior positions of pFST for some subjects (seeFig. 3, e.g., subject 0037), but more often found pFST ventral (e.g., subject 0248) to the other motion-selective areas. These differences may be attributed to individual variability in the precise position of pFST. Differences in our stereomotion protocols may provide further explanation for the difference in average position. First, our coherent stereomotion condition contained two disparity-defined (toward/away) surfaces for which disparity increased (or decreased). We used disparity-defined surfaces based on evidence that FST responds strongly to surfaces such as motion-defined 3D shapes (Mysore et al., 2010) and that selective processing of shapes/objects occurs ventral to hMT/MST (Kourtzi et al., 2002). Second, our control condition differed—our temporally scrambled condition contained several disparities (stimulus elements at different depths). Altogether, their study and ours both support the hypothesis that processing of stereomotion occurs adjacent to MT/MST. In addition, we both show that more complex visual-motion stimuli activate cortical areas that are: (1) not necessarily activated by 2D-motion localizers and (2) associated with more “downstream” processing within the visual hierarchy.

### 4.1 Population receptive field mapping

Retinotopic-mapping procedures are used to delineate several visual cortical regions based on their visual field maps, often by considering the eccentricity gradients and polar angle reversals that comprise a visual hemifield (or quarter field) representation (Dumoulin & Wandell, 2008;Wandell et al., 2007;Winawer & Witthoft, 2015). We found that the pFST boundaries were not reliably estimated using canonical retinotopic-mapping procedures. We conclude this for the following reasons. First, the parameter estimates of pRF eccentricity, polar angle, and size did not yield the common signatures of cortical retinotopic maps (e.g., full hemifield representations, or smooth gradients of size and/or visual-field position). Second, the explained variance tended to be quite low in these regions (less than 12%), making the pRF model estimates less reliable than for hMT/MST. Third, the pRFs were severely underestimated in size (<0.5 deg) and biased toward the center of the visual field. Receptive fields in the macaque inferior temporal region are, indeed, known to overrepresent the fovea (Desimone & Schein, 1987) but macaque FST, in particular, is known to have quite large RFs (~8–35 deg;Desimone & Ungerleider, 1986) at the eccentricities measured in this study. We then demonstrated how large, noisy RFs lead to poor estimates (with underestimated sizes and low explained variance). Similar biases have been previously commented on (Harvey & Dumoulin, 2011). Additionally, cortical regions anterior and ventral to hMT have similar variance explained using a pRF model and an ON-OFF contrast model (Benson et al., 2018), suggesting a lack of (or greatly reduced) spatial selectivity in those areas.

But why is the signal from the mapping procedure so unreliable for pFST? Historically, macaque FST has been considered non-topographic (Felleman & Van Essen, 1991) but a recent electrophysiology study showed a systematic change in size and eccentricity from posterior to anterior in FST (Thompson et al., 2023), suggesting a coarse topographic organization. If this region is topographically organized in humans, the lack of such evidence in our data is likely due to the constraints of our display in the MR-bore. The very large receptive-field size of neurons in FST would exceed our 12.2 deg stimulus radius, thus requiring a larger stimulus display range to obtain topographic gradients of the visual field. Several groups have retinotopically mapped human MT and surrounding regions using fMRI (Amano et al., 2009;Huk et al., 2002;Kolster et al., 2010). Kolster et al. used similar methods to identify a pFST region using retinotopic maps, largely relying on a shared foveal confluence between areas in the MT cluster. In this work, FST maps were very coarse and pRF size was smaller than in MT, potentially indicating a similar underestimation. Surprisingly, despite using a smaller stimulus aperture (7.75 vs. 12.2 deg radius), they found gradual changes in eccentricity and polar-angle estimates. They highlight a slow duty-cycle (64 s or greater) of the retinotopic stimulus as optimal for these regions.

Other studies have successfully mapped areas with large receptive fields using wide-field retinotopic mapping, such as V6 in humans (Pitzalis et al., 2006) and the posterior parietal cortex in monkeys (Rima et al., 2020). These studies suggest that expanding the stimulus beyond the conventional range used in fMRI experiments may be necessary to resolve the coarse topographic structure of FST. Altogether, it is possible that pFST has retinotopic organization albeit with a coarser spatial organization. To test this directly, the optimal stimuli would slowly sample space across a much larger window to obtain differential responses across neurons.

### 4.2 Functional selectivity of FST

Our localization of pFST should not be taken to indicate that this cortical area is exclusively dedicated to 3D-motion processing. There is speculation that the extrastriate body area (EBA) may overlap (at least partially) with FST. The EBA is known to consist of three non-contiguous areas (LOS/MOG, MTG, and ITG) surrounding hMT+ that are selectively activated by images of limbs (Weiner & Grill-Spector, 2011). The authors suggested that the limb-selective ITG likely overlaps with pFST. This is possible as human FST (localized by atlas) is activated by leg movements, unlike MT (Sulpizio et al., 2022). This is in line with macaque FST, which includes non-visual motion-responsive cells (Erickson et al., 1989). We can, therefore, expect that this area serves multiple functions across higher-level visual dimensions (Wurm & Caramazza, 2022). As a separate but related point, the pFST region is not the only region activated by our 3D-motion localizer. We have shown that hMT/MST is somewhat responsive to 3D motion as well, suggesting that cortical areas that process 2D and 3D motion are not mutually exclusive. The 2D-motion localizer tended to activate less of the lateral occipital cortex than the 3D-motion localizer, so we assumed that cortical areas that are activated by 3D- but not 2D-motion signals fall within FST.

### 4.3 Motion opponency

A critical question is how FST, which receives input from MT, can overcome the motion-opponency effect found in MT. Electrophysiological recordings in macaques have shown that only a small proportion (~10%) of FST neurons is direction selective, while a larger subset (~35%) is axially tuned, that is, responds to a given direction of motion, as well as motion in the opposite direction, without the opponency suppression seen in MT (Rosenberg et al., 2008). The model proposed byRosenberg et al. (2008)suggests that FST neurons compute a linear combination of the outputs from two pools of MT neurons tuned to opposite directions and different disparities. This integration allows FST to maintain robust responses to transparent motion by separating motion signals in different depth planes, thereby bypassing the suppression inherent to MT. Future work could explore developing a localizer based on contrasting between 3D-motion and opponent-motion stimuli for functionally localizing FST in humans, as motion opponency is a specific property of MT neurons that does not generalize to FST.

### 4.4 The human motion complex

Throughout this work, we refrained from making claims about whether FST lies within the human MT complex (hMT+). The MT region and its surrounding areas were initially termed a “complex” due to pending clarification, as it was understood that there were several motion-responsive areas forming a cluster in the macaque (including MT, MST, V4t, and FST;DeYoe et al., 1996). However, in the context of most human fMRI studies, hMT+ is operationalized as the cortical region responsive to moving versus static dots (2D-motion localizer), which surely includes MT and parts of MST, but may not include FST. To avoid giving too much credence to a term that is historically ambiguous, we instead focus on the ways that FST is functionally and structurally distinct from MT/MST. As delineation methods improve for motion-selective regions (e.g., based on functional specialization), the “complex” will either become a less useful term, or will need to be re-operationalized to make explicit what functions it includes. Our aim was also not to parcellate all possible areas of the MT complex. However, we wish to outline a potential subdivision that we did not address. In owl monkeys, FST is believed to include two distinct subareas, FSTd and FSTv (Kaas & Morel, 1993). V4t, another region associated with the complex, was not considered in our study due to a lack of research specifically addressing its functional and anatomical properties in humans.

### 4.5 From monkey to human

Our study was based on prior work in monkey, the most prominent animal model used for insight into human visual processing. Although there are several commonalities among primate species regarding motion processing (Orban et al., 2004), there are also important differences between humans and monkeys (Vanduffel et al., 2002). For example, motion area MT is in the middle temporal sulcus in the owl monkey (Allman & Kaas, 1971;Kaas & Morel, 1993), on the lateral surface of the marmoset (Spatz, 1977), in the superior temporal sulcus of the macaque (Gattass & Gross, 1981;Weller & Kaas, 1983), and in the inferior temporal sulcus of humans (Dumoulin et al., 2000). Prior human studies have also reported functional differences in putatively homologous visual areas compared to macaques. In particular,Armendariz et al. (2019)found depth-cue integration primarily within area MT in macaques, but predominantly within the V3B/KO area in humans. Similar species-specific functional divergence could potentially exist in FST and should be tested by future studies combining fMRI in humans and monkeys as well as neural recordings in macaques. Another possibility is that some of this variability may be related to challenges in functional parsing of the MT complex, as its precise boundaries and subdivision remain under investigation. Challenges in isolating distinct subregions within hMT+ could contribute to inconsistencies in reported responses to stereoscopic stimuli across studies.

### 4.6 Technical limitations and methodological considerations

The current study demonstrates that atlas-defined hMT/MST was generally consistent with the average functionally-defined hMT/MST, but this was not the case for pFST. When considering individual ROIs, these functionally-defined areas were often misaligned with atlas-defined ROIs. The misalignment was often more extreme for pFST. There is no*a priori*reason to anticipate a newly defined functional area to perfectly correspond to an existing atlas-based region, particularly in higher-level cortical areas. In this study, we chose the Glasser atlas as our atlas-based comparison, where regions were defined based on differential architecture, function, connectivity and topography. However, we registered the Glasser atlas back to a participant’s native space based on structural features only (e.g., cortical folds). Thus, differences between individual functional and atlas ROIs in this work may potentially reflect differences in anatomical- and functional-based ROI alignment. Consistent with this possibility, prior work has shown that V1 aligns well across hemispheres based on its distinct calcarine sulcus landmark, whereas extrastriate areas exhibit greater variability (Bridge, 2011). Similarly,Nieto-Castañón and Fedorenko (2012)highlighted that misalignment between functional activations and anatomical landmarks is common. As our understanding of cortical organization improves, atlases can be refined to better integrate functional distinctions and account for individual differences rather than assuming fixed anatomical boundaries.

We do not have definitive parameters for the best 2D-motion localizer or opponent-motion stimuli. Our versions of these stimuli were designed as an initial proposal that can be built upon in future studies. The exact parameters used for each of the fMRI protocols were grounded in previous work; however, there is no certainty that these parameters (dot size, speed, etc.) are optimal for activating FST. We limited the delineation of pFST based on the cortical responses to 2D- and 3D-motion localizers. Additional metrics including the protocols we used as validation criteria in this study (motion opponency, myelin, and anatomical priors such as size and position) could be used to refine the drawing of FST.

An alternative approach to identifying pFST would be a data-driven clustering method incorporating all available functional and structural metrics. While this could reveal functionally- and structurally-distinct regions, it is also less constrained, with degrees of freedom that may not directly aid in identifying a homologous FST in humans. Since prior research has not definitively established a human FST, we took a hypothesis-driven approach, using features known from monkey studies to specifically search for a homologous region in humans. Future studies could explore data-driven approaches once the existence of the area becomes more established. Additionally, individualized, automated thresholding methods similar to those proposed byGorgolewski et al. (2012)could further refine functional delineation.

## 5 Conclusions

Our study provides evidence for a distinct putative FST region in the human brain, characterized by unique functional and structural properties. FST likely plays a crucial role in the visual motion-processing network, particularly in integrating complex motion signals. It is involved in perceiving and interpreting 3D motion, which is vital for navigating and interacting with a dynamic environment. Additionally, FST’s role might extend beyond motion processing to include other sensory inputs, contributing to a more comprehensive understanding of spatial perception. Identifying the FST homolog in humans can facilitate translational research between primate species and advance the mapping of sensory processing related to motion perception. Future research should aim to refine localization methods and explore the broader implications of FST in sensory-guided actions.

## Supplementary Material

Supplementary Material

Supplementary Video 1

Supplementary Video 2

Supplementary Video 3

## Data Availability

Data needed to reproduce the results reported in this paper is available at (https://openneuro.org/datasets/ds006207/versions/1.0.0). Analysis code has been deposited on GitHub (https://github.com/putiw/FST_public). Any additional information required to reanalyze the data reported in this paper is available upon request.

## Supplementary Materials

Supplementary Figure S1. Individual participant activation maps for 2D- and 3D-motion localizers.

Supplementary Video S1. 2D-motion localizer stimulus video.

Supplementary Video S2. 3D-motion localizer stimulus video.

Supplementary Video S3. Opponent-motion stimulus video.

## References

[b1] Abdollahi , R. O. , Kolster , H. , Glasser , M. F. , Robinson , E. C. , Coalson , T. S. , Dierker , D. , Jenkinson , M. , Van Essen , D. C. , & Orban , G. A. ( 2014 ). Correspondences between retinotopic areas and myelin maps in human visual cortex . NeuroImage , 99 ( 100 ), 509 – 524 . 10.1016/j.neuroimage.2014.06.042 24971513 PMC4121090

[b2] Albright , T. D. ( 1984 ). Direction and orientation selectivity of neurons in visual area MT of the macaque . Journal of Neurophysiology , 52 ( 6 ), 1106 – 1130 . 10.1152/jn.1984.52.6.1106 6520628

[b3] Allman , J. M. , & Kaas , J. H. ( 1971 ). A representation of the visual field in the caudal third of the middle tempral gyrus of the owl monkey ( *Aotus trivirgatus* ) . Brain Research , 31 ( 1 ), 85 – 105 . 10.1016/0006-8993(71)90635-4 4998922

[b4] Amano , K. , Wandell , B. A. , & Dumoulin , S. O. ( 2009 ). Visual field maps, population receptive field sizes, and visual field coverage in the human MT+ complex . Journal of Neurophysiology , 102 ( 5 ), 2704 – 2718 . 10.1152/jn.00102.2009 19587323 PMC2777836

[b5] Annese , J. , Gazzaniga , M. S. , & Toga , A. W. ( 2005 ). Localization of the human cortical visual area MT based on computer aided histological analysis . Cerebral Cortex (New York, N.Y.: 1991) , 15 ( 7 ), 1044 – 1053 . 10.1093/cercor/bhh205 15590914

[b6] Armendariz , M. , Ban , H. , Welchman , A. E. , & Vanduffel , W. ( 2019 ). Areal differences in depth cue integration between monkey and human . PLoS Biology , 17 ( 3 ), e2006405 . 10.1371/journal.pbio.2006405 30925163 PMC6457573

[b7] Benson , N. C. , Jamison , K. W. , Arcaro , M. J. , Vu , A. T. , Glasser , M. F. , Coalson , T. S. , Van Essen , D. C. , Yacoub , E. , Ugurbil , K. , Winawer , J. , & Kay , K. ( 2018 ). The Human Connectome Project 7 Tesla retinotopy dataset: Description and population receptive field analysis . Journal of Vision , 18 ( 13 ), 23 . 10.1167/18.13.23 PMC6314247 30593068

[b8] Boussaoud , D. , Ungerleider , L. G. , & Desimone , R. ( 1990 ). Pathways for motion analysis: Cortical connections of the medial superior temporal and fundus of the superior temporal visual areas in the macaque . The Journal of Comparative Neurology , 296 ( 3 ), 462 – 495 . 10.1002/cne.902960311 2358548

[b9] Brainard , D. H. ( 1997 ). The Psychophysics Toolbox . Spatial Vision , 10 ( 4 ), 433 – 436 . https://www.ncbi.nlm.nih.gov/pubmed/9176952 9176952

[b10] Bridge , H. ( 2011 ). Mapping the visual brain: How and why . Eye , 25 ( 3 ), 291 - 296 . 10.1038/eye.2010.166 21102491 PMC3178304

[b11] Clarke , S. , & Miklossy , J. ( 1990 ). Occipital cortex in man: Organization of callosal connections, related myelo- and cytoarchitecture, and putative boundaries of functional visual areas . The Journal of Comparative Neurology , 298 ( 2 ), 188 – 214 . 10.1002/cne.902980205 2212102

[b12] Cléry , J. C. , Schaeffer , D. J. , Hori , Y. , Gilbert , K. M. , Hayrynen , L. K. , Gati , J. S. , Menon , R. S. , & Everling , S. ( 2020 ). Looming and receding visual networks in awake marmosets investigated with fMRI . NeuroImage , 215 , 116815 . 10.1016/j.neuroimage.2020.116815 32278898

[b13] Czuba , T. B. , Huk , A. C. , Cormack , L. K. , & Kohn , A. ( 2014 ). Area MT encodes three-dimensional motion . The Journal of Neuroscience: The Official Journal of the Society for Neuroscience , 34 ( 47 ), 15522 – 15533 . 10.1523/JNEUROSCI.1081-14.2014 25411482 PMC4236390

[b14] Desimone , R. , & Schein , S. J. ( 1987 ). Visual properties of neurons in area V4 of the macaque: Sensitivity to stimulus form . Journal of Neurophysiology , 57 ( 3 ), 835 – 868 . 10.1152/jn.1987.57.3.835 3559704

[b15] Desimone , R. , & Ungerleider , L. G. ( 1986 ). Multiple visual areas in the caudal superior temporal sulcus of the macaque . The Journal of Comparative Neurology , 248 ( 2 ), 164 – 189 . 10.1002/cne.902480203 3722457 PMC11528348

[b16] DeYoe , E. A. , Carman , G. J. , Bandettini , P. , Glickman , S. , Wieser , J. , Cox , R. , Miller , D. , & Neitz , J. ( 1996 ). Mapping striate and extrastriate visual areas in human cerebral cortex . Proceedings of the National Academy of Sciences of the United States of America , 93 ( 6 ), 2382 – 2386 . 10.1073/pnas.93.6.2382 8637882 PMC39805

[b17] Dubner , R. , & Zeki , S. M. ( 1971 ). Response properties and receptive fields of cells in an anatomically defined region of the superior temporal sulcus in the monkey . Brain Research , 35 ( 2 ), 528 – 532 . 10.1016/0006-8993(71)90494-x 5002708

[b18] Dumoulin , S. O. , Bittar , R. G. , Kabani , N. J. , Baker , C. L. , Jr , Le Goualher , G. , Bruce Pike , G., & Evans , A. C. ( 2000 ). A new anatomical landmark for reliable identification of human area V5/MT: A quantitative analysis of sulcal patterning . Cerebral Cortex (New York, N.Y.: 1991) , 10 ( 5 ), 454 – 463 . 10.1093/cercor/10.5.454 10847595

[b19] Dumoulin , S. O. , & Wandell , B. A. ( 2008 ). Population receptive field estimates in human visual cortex . NeuroImage , 39 ( 2 ), 647 – 660 . 10.1016/j.neuroimage.2007.09.034 17977024 PMC3073038

[b20] Erickson , R. G. , Dow , B. M. , & Snyder , A. Z. ( 1989 ). Representation of the fovea in the superior temporal sulcus of the macaque monkey . Experimental Brain Research , 78 ( 1 ), 90 – 112 . 10.1007/BF00230690 2591521

[b21] Esteban , O. , Markiewicz , C. J. , Blair , R. W. , Moodie , C. A. , Isik , A. I. , Erramuzpe , A. , Kent , J. D. , Goncalves , M. , DuPre , E. , Snyder , M. , Oya , H. , Ghosh , S. S. , Wright , J. , Durnez , J. , Poldrack , R. A. , & Gorgolewski , K. J. ( 2019 ). fMRIPrep: A robust preprocessing pipeline for functional MRI . Nature Methods , 16 ( 1 ), 111 – 116 . 10.1038/s41592-018-0235-4 30532080 PMC6319393

[b22] Felleman , D. J. , & Van Essen , D. C. ( 1991 ). Distributed hierarchical processing in the primate cerebral cortex . Cerebral Cortex (New York, N.Y.: 1991) , 1 ( 1 ), 1 – 47 . 10.1093/cercor/1.1.1-a 1822724

[b150] Fox , C. J. , Iaria , G. , & Barton , J. J. ( 2009 ). Defining the face processing network: Optimization of the functional localizer in fMRI . Human Brain Mapping , 30 ( 5 ), 1637 – 1651 . 10.1002/hbm.20630 18661501 PMC6870735

[b23] Gattass , R. , & Gross , C. G. ( 1981 ). Visual topography of striate projection zone (MT) in posterior superior temporal sulcus of the macaque . Journal of Neurophysiology , 46 ( 3 ), 621 – 638 . 10.1152/jn.1981.46.3.621 7299437

[b24] Glasser , M. F. , Coalson , T. S. , Robinson , E. C. , Hacker , C. D. , Harwell , J. , Yacoub , E. , Ugurbil , K. , Andersson , J. , Beckmann , C. F. , Jenkinson , M. , Smith , S. M. , & Van Essen , D. C. ( 2016 ). A multi-modal parcellation of human cerebral cortex . Nature , 536 ( 7615 ), 171 – 178 . 10.1038/nature18933 27437579 PMC4990127

[b25] Gorgolewski , K. J. , Alfaro-Almagro , F. , Auer , T. , Bellec , P. , Capotă , M. , Chakravarty , M. M. , Churchill , N. W. , Cohen , A. L. , Craddock , R. C. , Devenyi , G. A. , Eklund , A. , Esteban , O. , Flandin , G. , Ghosh , S. S. , Guntupalli , J. S. , Jenkinson , M. , Keshavan , A. , Kiar , G. , Liem , F. , … Poldrack , R. A. ( 2017 ). BIDS apps: Improving ease of use, accessibility, and reproducibility of neuroimaging data analysis methods . PLoS Computational Biology , 13 ( 3 ), e1005209 . 10.1371/journal.pcbi.1005209 28278228 PMC5363996

[b151] Gorgolewski , K. J. , Storkey , A. J. , Bastin , M. E. , & Pernet , C. R. ( 2012 ). Adaptive thresholding for reliable topological inference in single subject fMRI analysis . Frontiers in Human Neuroscience , 6 , 245 . 10.3389/fnhum.2012.00245 22936908 PMC3427544

[b26] Harvey , B. M. , & Dumoulin , S. O. ( 2011 ). The relationship between cortical magnification factor and population receptive field size in human visual cortex: Constancies in cortical architecture . The Journal of Neuroscience: The Official Journal of the Society for Neuroscience , 31 ( 38 ), 13604 – 13612 . 10.1523/JNEUROSCI.2572-11.2011 21940451 PMC6623292

[b27] Heeger , D. J. , Boynton , G. M. , Demb , J. B. , Seidemann , E. , & Newsome , W. T. ( 1999 ). Motion opponency in visual cortex . The Journal of Neuroscience: The Official Journal of the Society for Neuroscience , 19 ( 16 ), 7162 – 7174 . 10.1523/JNEUROSCI.19-16-07162.1999 10436069 PMC6782843

[b28] Héjja-Brichard , Y. , Rima , S. , Rapha , E. , Durand , J.-B. , & Cottereau , B. R. ( 2020 ). Stereomotion processing in the nonhuman primate brain . Cerebral Cortex , 30 ( 8 ), 4528 – 4543 . 10.1093/cercor/bhaa055 32227117

[b29] Himmelberg , M. M. , Winawer , J. , & Carrasco , M. ( 2022 ). Linking individual differences in human primary visual cortex to contrast sensitivity around the visual field . Nature Communications , 13 ( 1 ), 3309 . 10.1038/s41467-022-31041-9 PMC9192713 35697680

[b30] Huk , A. C. , Dougherty , R. F. , & Heeger , D. J. ( 2002 ). Retinotopy and functional subdivision of human areas MT and MST . The Journal of Neuroscience: The Official Journal of the Society for Neuroscience , 22 ( 16 ), 7195 – 7205 . 10.1523/JNEUROSCI.22-16-07195.2002 12177214 PMC6757870

[b31] Kaas , J. H. , & Morel , A. ( 1993 ). Connections of visual areas of the upper temporal lobe of owl monkeys: The MT crescent and dorsal and ventral subdivisions of FST . The Journal of Neuroscience: The Official Journal of the Society for Neuroscience , 13 ( 2 ), 534 – 546 . 10.1523/JNEUROSCI.13-02-00534.1993 8381166 PMC6576646

[b32] Karakuzu , A. , Boudreau , M. , Duval , T. , Boshkovski , T. , Leppert , I. , Cabana , J. F. , Gagnon , I. , Beliveau , P. , Pike , G. B. , Cohen-Adad , J. , & Stikov , N. ( 2020 ). qMRLab: Quantitative MRI analysis, under one umbrella . Journal of Open Source Software , 5 ( 53 ), 2343 . 10.21105/joss.02343

[b33] Kleiner , M. , Brainard , D. , Pelli , D. , Ingling , A. , Murray , R. , & Broussard , C. ( 2007 ). What’s new in psychtoolbox-3 . Perception , 36 ( 14 ), 1 – 16 . https://hdl.handle.net/11858/00-001M-0000-0013-CC89-F

[b34] Kolster , H. , Mandeville , J. B. , Arsenault , J. T. , Ekstrom , L. B. , Wald , L. L. , & Vanduffel , W. ( 2009 ). Visual field map clusters in macaque extrastriate visual cortex . The Journal of Neuroscience: The Official Journal of the Society for Neuroscience , 29 ( 21 ), 7031 – 7039 . 10.1523/JNEUROSCI.0518-09.2009 19474330 PMC2749229

[b35] Kolster , H. , Peeters , R. , & Orban , G. A. ( 2010 ). The retinotopic organization of the human middle temporal area MT/V5 and its cortical neighbors . The Journal of Neuroscience: The Official Journal of the Society for Neuroscience , 30 ( 29 ), 9801 – 9820 . 10.1523/JNEUROSCI.2069-10.2010 20660263 PMC6632824

[b36] Kourtzi , Z. , Bülthoff , H. H. , Erb , M. , & Grodd , W. ( 2002 ). Object-selective responses in the human motion area MT/MST . Nature Neuroscience , 5 ( 1 ), 17 – 18 . 10.1038/nn780 11740503

[b37] Krubitzer , L. A. , & Kaas , J. H. ( 1990 ). Cortical connections of MT in four species of primates: Areal, modular, and retinotopic patterns . Visual Neuroscience , 5 ( 2 ), 165 – 204 . 10.1017/s0952523800000213 2278944

[b38] Likova , L. T. , & Tyler , C. W. ( 2007 ). Stereomotion processing in the human occipital cortex . NeuroImage , 38 ( 2 ), 293 – 305 . 10.1016/j.neuroimage.2007.06.039 17869540

[b39] Mancini , M. , Karakuzu , A. , Cohen-Adad , J. , Cercignani , M. , Nichols , T. E. , & Stikov , N. ( 2020 ). An interactive meta-analysis of MRI biomarkers of myelin . eLife , 9 , e61523 . 10.7554/elife.61523 33084576 PMC7647401

[b40] Maunsell , J. H. , & Van Essen , D. C. ( 1983a ). Functional properties of neurons in middle temporal visual area of the macaque monkey. I. Selectivity for stimulus direction, speed, and orientation . Journal of Neurophysiology , 49 ( 5 ), 1127 – 1147 . 10.1152/jn.1983.49.5.1127 6864242

[b41] Maunsell , J. H. , & Van Essen , D. C. ( 1983b ). Functional properties of neurons in middle temporal visual area of the macaque monkey. II. Binocular interactions and sensitivity to binocular disparity . Journal of Neurophysiology , 49 ( 5 ), 1148 – 1167 . 10.1152/jn.1983.49.5.1148 6864243

[b42] Mysore , S. G. , Vogels , R. , Raiguel , S. E. , Todd , J. T. , & Orban , G. A. ( 2010 ). The selectivity of neurons in the macaque fundus of the superior temporal area for three-dimensional structure from motion . The Journal of Neuroscience: The Official Journal of the Society for Neuroscience , 30 ( 46 ), 15491 – 15508 . 10.1523/JNEUROSCI.0820-10.2010 21084605 PMC6633687

[b43] Nelissen , K. , Vanduffel , W. , & Orban , G. A. ( 2006 ). Charting the lower superior temporal region, a new motion-sensitive region in monkey superior temporal sulcus . The Journal of Neuroscience: The Official Journal of the Society for Neuroscience , 26 ( 22 ), 5929 – 5947 . 10.1523/JNEUROSCI.0824-06.2006 16738235 PMC6675228

[b44] Nieto-Castañón , A. , & Fedorenko , E. ( 2012 ). Subject-specific functional localizers increase sensitivity and functional resolution of multi-subject analyses . NeuroImage , 63 ( 3 ), 1646 – 1669 . 10.1016/j.neuroimage.2012.06.065 22784644 PMC3477490

[b45] Orban , G. A. , Van Essen , D. , & Vanduffel , W. ( 2004 ). Comparative mapping of higher visual areas in monkeys and humans . Trends in Cognitive Sciences , 8 ( 7 ), 315 – 324 . 10.1016/j.tics.2004.05.009 15242691

[b46] Pelli , D. G. ( 1997 ). The VideoToolbox software for visual psychophysics: Transforming numbers into movies . Spatial Vision , 10 ( 4 ), 437 – 442 . https://www.ncbi.nlm.nih.gov/pub med/9176953 9176953

[b47] Pitzalis , S. , Galletti , C. , Huang , R. S. , Patria , F. , Committeri , G. , Galati , G. , Fattori , P. , & Sereno , M. I. ( 2006 ). Wide-field retinotopy defines human cortical visual area V6 . Journal of Neuroscience , 26 ( 30 ), 7962 – 7973 . 10.1523/jneurosci.0178-06.2006 16870741 PMC6674231

[b48] Rima , S. , Cottereau , B. R. , Héjja-Brichard , Y. , Trotter , Y. , & Durand , J. B. ( 2020 ). Wide-field retinotopy reveals a new visuotopic cluster in macaque posterior parietal cortex . Brain Structure and Function , 225 , 2447 – 2461 . 10.1007/s00429-020-02134-2 32875354 PMC7544618

[b49] Qian , N. , & Andersen , R. A. ( 1994 ). Transparent motion perception as detection of unbalanced motion signals. II. Physiology . The Journal of Neuroscience: The Official Journal of the Society for Neuroscience , 14 ( 12 ), 7367 – 7380 . 10.1523/JNEUROSCI.14-12-07367.1994 7996182 PMC6576905

[b50] Rokers , B. , Cormack , L. K. , & Huk , A. C. ( 2009 ). Disparity- and velocity-based signals for three-dimensional motion perception in human MT+ . Nature Neuroscience , 12 ( 8 ), 1050 – 1055 . 10.1038/nn.2343 19578382

[b51] Rosa , M. G. , Soares , J. G. , Fiorani , M. , Jr , & Gattass , R. ( 1993 ). Cortical afferents of visual area MT in the Cebus monkey: Possible homologies between New and Old World monkeys . Visual Neuroscience , 10 ( 5 ), 827 – 855 . 10.1017/s0952523800006064 8217935

[b52] Rosenberg , A. , Thompson , L. W. , Doudlah , R. , & Chang , T.-Y. ( 2023 ). Neuronal representations supporting three-dimensional vision in nonhuman primates . Annual Review of Vision Science , 9 , 337 – 359 . 10.1146/annurev-vision-111022-123857 36944312

[b53] Rosenberg , A. , Wallisch , P. , & Bradley , D. C. ( 2008 ). Responses to direction and transparent motion stimuli in area FST of the macaque . Visual Neuroscience , 25 ( 2 ), 187 – 195 . 10.1017/S0952523808080528 18442441

[b54] Saito , H. , Yukie , M. , Tanaka , K. , Hikosaka , K. , Fukada , Y. , & Iwai , E. ( 1986 ). Integration of direction signals of image motion in the superior temporal sulcus of the macaque monkey . The Journal of Neuroscience: The Official Journal of the Society for Neuroscience , 6 ( 1 ), 145 – 157 . 10.1523/JNEUROSCI.06-01-00145.1986 3944616 PMC6568620

[b55] Sanada , T. M. , & DeAngelis , G. C. ( 2014 ). Neural representation of motion-in-depth in area MT . The Journal of Neuroscience: The Official Journal of the Society for Neuroscience , 34 ( 47 ), 15508 – 15521 . 10.1523/JNEUROSCI.1072-14.2014 25411481 PMC4236389

[b56] Spatz , W. B. ( 1977 ). Topographically organized reciprocal connections between areas 17 and MT (visual area of superior temporal sulcus) in the marmoset *Callithrix jacchus* . Experimental Brain Research , 27 ( 5 ), 559 – 572 . 10.1007/BF00239044 404175

[b300] Stevens , M. T. R. , D’Arcy , R. C. , Stroink , G. , Clarke , D. B. , & Beyea , S. D. ( 2013 ). Thresholds in fMRI studies: reliable for single subjects? Journal of Neuroscience Methods , 219 ( 2 ), 312 – 323 . 10.1016/j.jneumeth.2013.08.005 23958749

[b57] Sulpizio , V. , Strappini , F. , Fattori , P. , Galati , G. , Galletti , C. , Pecchinenda , A. , & Pitzalis , S. ( 2022 ). The human middle temporal cortex responds to both active leg movements and egomotion-compatible visual motion . Brain Structure & Function , 227 ( 8 ), 2573 – 2592 . 10.1007/s00429-022-02549-z 35963915

[b58] Thompson , L. W. , Kim , B. , Rokers , B. , & Rosenberg , A. ( 2023 ). Hierarchical computation of 3D motion across macaque areas MT and FST . Cell Reports , 42 ( 12 ), 113524 . 10.1016/j.celrep.2023.113524 38064337 PMC10791528

[b59] Tootell , R. B. , Reppas , J. B. , Dale , A. M. , Look , R. B. , Sereno , M. I. , Malach , R. , Brady , T. J. , & Rosen , B. R. ( 1995 ). Visual motion aftereffect in human cortical area MT revealed by functional magnetic resonance imaging . Nature , 375 ( 6527 ), 139 – 141 . 10.1038/375139a0 7753168

[b60] Ungerleider , L. G. , & Desimone , R. ( 1986a ). Cortical connections of visual area MT in the macaque . The Journal of Comparative Neurology , 248 ( 2 ), 190 – 222 . 10.1002/cne.902480204 3722458

[b61] Ungerleider , L. G. , & Desimone , R. ( 1986b ). Projections to the superior temporal sulcus from the central and peripheral field representations of V1 and V2 . The Journal of Comparative Neurology , 248 ( 2 ), 147 – 163 . 10.1002/cne.902480202 3722456

[b62] Vanduffel , W. , Fize , D. , Peuskens , H. , Denys , K. , Sunaert , S. , Todd , J. T. , & Orban , G. A. ( 2002 ). Extracting 3D from motion: Differences in human and monkey intraparietal cortex . Science , 298 ( 5592 ), 413 – 415 . 10.1126/science.1073574 12376701

[b63] Van Essen , D. C. , Maunsell , J. H. , & Bixby , J. L. ( 1981 ). The middle temporal visual area in the macaque: Myeloarchitecture, connections, functional properties and topographic organization . The Journal of Comparative Neurology , 199 ( 3 ), 293 – 326 . 10.1002/cne.901990302 7263951

[b64] Wandell , B. A. , Dumoulin , S. O. , & Brewer , A. A. ( 2007 ). Visual field maps in human cortex . Neuron , 56 ( 2 ), 366 – 383 . 10.1016/j.neuron.2007.10.012 17964252

[b65] Wansapura , J. P. , Holland , S. K. , Dunn , R. S. , & Ball , W. S. , Jr. ( 1999 ). NMR relaxation times in the human brain at 3.0 tesla . Journal of Magnetic Resonance Imaging: JMRI , 9 ( 4 ), 531 – 538 . 10.1002/(sici)1522-2586(199904)9:4<531::aid-jmri4>3.0.co;2-l 10232510

[b66] Weiner , K. S. , & Grill-Spector , K. ( 2011 ). Not one extrastriate body area: Using anatomical landmarks, hMT+, and visual field maps to parcellate limb-selective activations in human lateral occipitotemporal cortex . NeuroImage , 56 ( 4 ), 2183 – 2199 . 10.1016/j.neuroimage.2011.03.041 21439386 PMC3138128

[b67] Weller , R. E. , & Kaas , J. H. ( 1983 ). Retinotopic patterns of connections of area 17 with visual areas V-II and MT in macaque monkeys . The Journal of Comparative Neurology , 220 ( 3 ), 253 – 279 . 10.1002/cne.902200302 6315784

[b68] Wen , P. , Landy , M. S. , & Rokers , B. ( 2023 ). Identifying cortical areas that underlie the transformation from 2D retinal to 3D head-centric motion signals . NeuroImage , 270 , 119909 . 10.1016/j.neuroimage.2023.119909 36801370 PMC10061442

[b69] Winawer , J. , & Witthoft , N. ( 2015 ). Human V4 and ventral occipital retinotopic maps . Visual Neuroscience , 32 , E020 . 10.1017/S0952523815000176 26241699 PMC4730874

[b70] Wurm , M. F. , & Caramazza , A. ( 2022 ). Two “what” pathways for action and object recognition . Trends in Cognitive Sciences , 26 ( 2 ), 103 – 116 . 10.1016/j.tics.2021.10.003 34702661

