# Supplementary material for: Functional localization of visual motion area FST in humans
Source: Imaging Neurosci (Camb). 2025 May 16;3:imag_a_00578. doi: 10.1162/imag_a_00578 (PMC12162087; doi:10.1162/imag_a_00578)
Supplement: Supplementary Material [file imag_a_00578-supp.pdf]

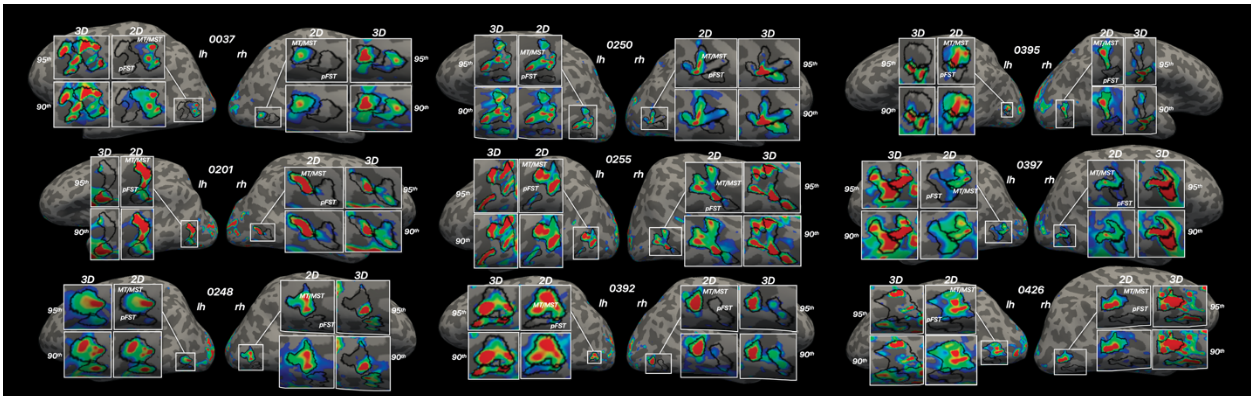

Supplementary Figure 1. Individual participant activation maps for 2D and 3D motion localizers. Activation maps for 2D- and 3D-motion responses are shown for each participant across both hemispheres (lh = left hemisphere, rh = right hemisphere). Each participant's data are presented with activation thresholds at both the 95th (top row of each panel) and 90th percentile (bottom row of each panel) to illustrate individual variability in functional responses.
